# Analysis of the Fatality Rate in Relation to Testing Capacity during the First 50 days of the COVID-19 Epidemic in Italy

**DOI:** 10.4269/ajtmh.20-0862

**Published:** 2020-10-15

**Authors:** Costanza Vicentini, Stefano Bazzolo, Dario Gamba, Carla Maria Zotti

**Affiliations:** 1 Department of Public Health and Paediatrics, University of Turin, Turin, Italy;; 2 Department of Environment, Land and Infrastructure Engineering (DIATI), Politecnico of Turin, Turin, Italy;; 3 Scuola di Medicina, Università of Turin, Turin, Italy

## Abstract

Italy has been one of the most severely affected countries by the COVID-19 pandemic, and the case fatality rate (CFR) estimated based on Italian data is one of the highest worldwide. We analyzed public data from the first 50 days of the epidemic in Italy (from February 24 to April 13, 2020) to evaluate whether evolving testing strategies and capacity could account for trends in the CFR. The CFR increased during the study period, and a significant positive correlation was found between the CFR and the percentage of positive tests among performed real-time PCR tests (positive tests % [POS%]) until March 25, suggesting the surveillance system did not detect a growing number of cases in the initial phase of the epidemic. To avoid distortion due to the delay between the identification of cases and deaths, the expected CFR (expCFR) was calculated, which represents the ratio between the predicted number of cases and deaths at the end of the epidemic based on the best fitting logistic curves of the cumulative numbers of cases and deaths. The expCFR began a downward trend from the 40th day. In the final phase, a decrease in both expCFR and POS% was identified, suggesting an improvement in surveillance. The results of this study suggest data from the first 50 days of the COVID-19 epidemic in Italy were severely affected by ascertainment bias. Insufficient testing and isolation of cases could have facilitated the widespread transmission of COVID-19 in the early stages of the outbreak.

## INTRODUCTION

The current COVID-19 pandemic has spread extensively, causing more than 12 million cases and 566,654 deaths globally as of July 14, 2020.^1^ Italy has been one of the most severely affected countries, with the fourth highest death toll worldwide. As of July 14, 2020, more than 240,000 cases and 30,000 deaths related to COVID-19 have been identified,^2^ with important variations among Italian regions and a strong north to south gradient.^3^ The most severely affected regions were those in which the earliest outbreaks occurred, although all regions recorded cases and deaths due to COVID-19 by the end of March 2020.^3,4^ The region of Lombardy, where one of the two initial outbreaks occurred,^5^ remains the Italian region with the highest burden of disease, accounting for 39.1% of the cumulative number of cases and 49.2% of deaths as of July 7, 2020.^6^

A crude case fatality rate (CFR) of 10.6% and 18.3% was estimated based on data from Italy and Lombardy, respectively,^5^ which is much higher than what would be expected according to data from the Chinese outbreak (3.6%) or from the Diamond Princess cruise ship, where robust denominator data were available as all infections were identified (1%).^7,8^ Several hypotheses have been suggested to explain the higher lethality of COVID-19 in Italy, such as the older age distribution, differences in the definition of deaths related to COVID-19 compared with other countries, evolving testing strategies,^9^ and in the case of Lombardy, the breakdown of the National Health Service due to the dramatic surge in cases.^5^

The assessment of the CFR of an ongoing outbreak, although critical to guide public health interventions, can be affected by several biases. Key clinical and epidemiologic characteristics of the disease are still unknown. Right censoring, caused by the delay between symptom onset and death between vulnerable and healthy individuals, and ascertainment bias, due to underreporting of asymptomatic and mildly symptomatic infections, can, respectively, lead to under- and overestimations of the CFR.^10^ In the early stages of the outbreak, an evaluation of case exportations from Italy between February 25 and 29, 2020, suggested 27–75% of cases in the country had not been identified.^11^ Furthermore, because of the regional structure of the Italian National Health Service, testing strategies and laboratory capacity differed greatly across regions, ultimately leading to inhomogeneous data.^5,12^

In this study, data from the COVID-19 surveillance were analyzed to interpret the evolution of the first 50 days of the epidemic in Italy. We investigated the trend in the CFR and assessed the correlation between fatality and percentage of positive tests among performed tests to evaluate whether evolving testing strategies and capacity could account for variations in the CFR.

## MATERIALS AND METHODS

### Study design and data sources.

Data collated by the Italian Civil Protection and made public by the Ministry of Health were used for this study. The Civil Protection’s online platform^2^ has been providing data on the outbreak in Italy since February 21, 2020, on a daily basis. Reported data include cumulative counts and daily increases in the number of performed tests, cases, hospitalized patients, patients hospitalized in intensive care units, and deaths, by Italian region and province. All data are de-identified and publicly available; therefore, no patient consent or ethics approval was required for this study.

For the purpose of this analysis, data from the region of Lombardy were considered separately from the rest of Italy because of the dramatic increase in the number of cases in the region which caused the health system to quickly reach maximum capacity.^13^ Data on cases reported from February 24, 2020 until April 13, 2020 were analyzed for the current study.

### Definitions and indications for testing.

As the outbreak rapidly spread across Italy, evolving case definitions and indications for testing were issued by the Ministry of Health. From February 25, testing was indicated for symptomatic patients with acute respiratory distress syndrome; severe acute respiratory infection (SARI); influenza-like illness without an alternative diagnosis and epidemiologically linked to areas with secondary transmission of COVID-19; and suspected cases, that is, patients with acute respiratory infection (ARI) and with either history of travel to areas reporting community transmission of COVID-19, close contact with a confirmed case, or, in the case of healthcare workers, having operated in an institution with confirmed cases, during the 14 days before symptom onset.^14^ On March 9, the definition of suspected case was updated to include all patients with ARI admitted to emergency rooms or seeking primary care in areas with evidence of community transmission of COVID-19.^15^ On April 3, the following were defined as a priority for testing: hospitalized patients with SARI, patients with ARI in hospitals or nursing homes, healthcare workers exposed to COVID-19 infection risk, symptomatic essential workers, patients at risk of developing severe disease, and first symptomatic individuals in closed communities.^16^

Concerning the definition of COVID-19 cases, in the early stages of the epidemic, for a case to be registered, two positive real-time PCR (RT-PCR) tests of respiratory specimens were required, as the diagnostic test performed at regional reference laboratories had to be confirmed by the National Institute of Health (Istituto Superiore di Sanità [ISS]).^14^ From March 9, 2020, in regions with sustained community transmission, confirmation by the ISS is no longer required.^15^ In Italy, all deaths occurring in patients with a positive RT-PCR test are identified as deaths related to COVID-19, regardless of underlying medical conditions or if the infection was the ultimate cause of death.^17^

### Statistical modeling.

A nonnegative logistic function was fitted on the cumulative number of cases and deaths as a function of time:

$$f\left(t\right)=\;\underset{t}{\max}\left(0;A^{*}\frac{1+B^{*}e^{\frac{-t}{C}}}{1+D^{*}e^{\frac{-t}{C}}}\;\right).$$

The constrain of nonnegativity was necessary to avoid obtaining negative theoretical values in the first days of the epidemic. Parameters *A*, *B*, *C*, and *D* were determined based on the best fit model of nonlinear regression with the Levenberg–Marqardt algorithm. To verify the solidity of the model, a χ^2^ test was performed on the absolute frequencies of the theoretical and observed data at 10 equal intervals.

The analysis of the best-fit parameters was interpolated daily to evaluate the trend as a function of time of the values of parameter A, which represents the horizontal asymptote of the function, that is, the predicted number of cases and deaths at the end of the epidemic. The parameters A, B, C, and D of the logistic curve were fitted daily, and their value was set each day according to the best fit models.

### Epidemiological parameters.

The CFR is traditionally calculated by dividing the number of deaths in patients who tested positive for COVID-19 by the cumulative number of cases at a specific point in time.^9^ Because of the delay between a positive test result and death, this measure is an underestimation of disease fatality when the calculation is not performed ex post.^10^ To avoid this distortion, we calculated the expected CFR (expCFR), expressed as the ratio between the A parameters of the best-fitting logistic curves of the cumulative number of deaths and cases, which represent the predicted number of cases and deaths at the end of the epidemic, interpolated at a specific point in time. Therefore, the calculation of the expCFR is not distorted by the time delay existing between the curves of the cumulative numbers of cases and deaths, which affects the calculation of the CFR.

The percentage of positive results among performed RT-PCR tests (POS%) and the number of performed RT-PCR tests were evaluated to assess the relationship between the number of cases and testing capacity, and to assess the ability of the Italian health system to respond to the increase in the number of cases with a proportional increase in the number of performed tests. We assumed the number of tests performed at the end of February, when the number of cases in Italy was limited, to have been sufficient to measure the scale of the epidemic. To continue to accurately monitor the epidemic, the increase in the number of positive tests (i.e., cases) should have been mirrored by a proportional increase in percentage terms in the number of performed tests.

We defined test variation % (TVAR%) as the daily percentage variation of the number of performed tests, divided by the daily percentage variation of POS%, as follows:

$$\mathrm{TVAR}\%=\frac{\Delta \text{TEST\%}}{\Delta \text{POS\%}}.$$

With ΔTEST% defined as the daily percentage variation of performed tests:

$$\Delta \text{TEST\%}=\frac{\mathrm{TES}T_{t}-\mathrm{TES}T_{t-1}}{\mathrm{TES}T_{t-1}}$$

and ΔPOS% defined as the daily percentage variation of POS%:

$$\Delta \text{POS\%}=\frac{\mathrm{POS}{\%}_{t}-\mathrm{POS}{\%}_{t-1}}{\mathrm{POS}{\%}_{t-1}}.$$

If the value of TVAR% is higher than 100%, the number of performed tests is increasing faster than POS%, whereas if the value is lower than 100%, the increase in testing capacity is not able to follow the increase of POS%.

### Correlation between fatality rate and POS%.

Scatterplots were used to assess the correlation between CFR and POS%, and expCFR and POS%. Analyses were performed using MINITAB 19 and MATLAB version R2020a (The MathWorks, Inc., Natick, MA).

## RESULTS

### Modeling cases and deaths.

**Figure 1.**
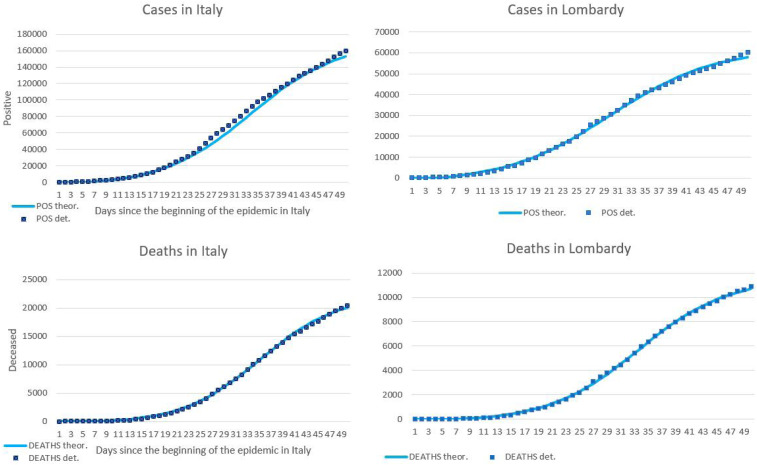
Cumulative number of COVID-19 cases and deaths registered in Italy and Lombardy from February 24 to April 13, 2020, and best-fitting logistic curves. (**A**) Cases in Italy. (**B**) Cases in Lombardy. (**C**) Deaths in Italy. (**D**) Deaths in Lombardy.

The best-fit values for the parameters of the nonnegative logistic functions modeling the cumulative number of cases and deaths are summarized in Table 1. Figure 1 shows the cumulative number of cases and deaths registered in Italy and Lombardy, and the best-fitting logistic curves. The *P*-value of the χ^2^ test performed on the absolute frequencies of the theoretical and observed data segmented in 10 equal intervals was < 0.001 for cases and 0.026 for deaths, supporting the validity of the model.

**Table 1:** Estimated values and standard errors of the parameters of the best-fitting logistic functions modeling the cumulative number of cases and deaths as a function of time, based on data from the 2020 COVID-19 outbreak in Italy and Lombardy

	Italy		Lombardy	
Parameter	Cases	Deaths	Cases	Deaths
A	170,785 (2,345.72)	22,193.2 (226.73)	62,441 (785)	11,559 (88)
B	−2.12 (0.25)	−3.4 (0.41)	−1.82 (0.19)	−3.04 (0.35)
C	7.54 (0.21)	6.63 (0.13)	7.71 (0,24)	6.4 (0.11)
D	77.35 (8.15)	205.94 (17.71)	48.39 (5.27)	180.37 (13.55)

The theoretical peak (i.e., the day on which the second-order derivative of the cumulative curves calculated considering the last fitting values takes a value of 0) was reached on March 27 and 24 for the curve of the cumulative number of cases, and March 29 and 28 for the curve of the cumulative number of deaths, respectively, for Italy and Lombardy. The actual peak (i.e., the day of maximum daily increase) was reached on March 21 for the curves of the cumulative number of cases of both Italy and Lombardy, and March 27 and 21 for the curve of the cumulative number of deaths, respectively, for Italy and Lombardy. This difference could be explained by an insufficient number of tests performed on the days of the actual peaks.

**Figure 2.**
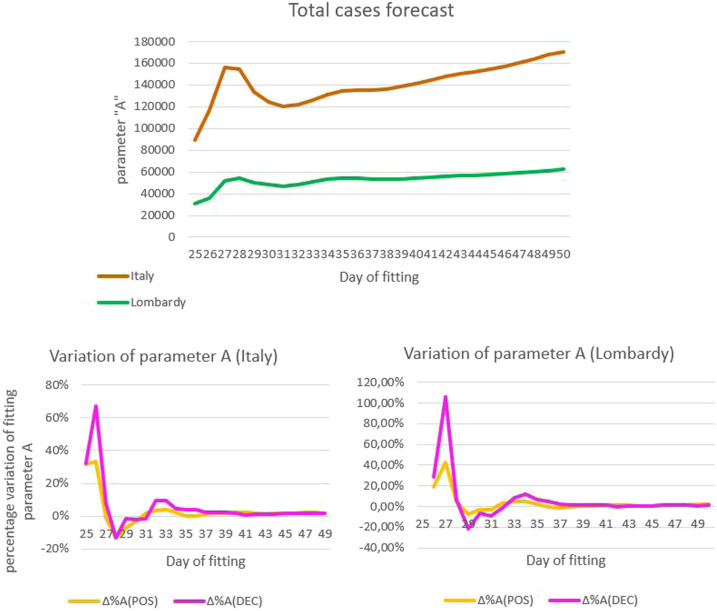
Trend in the values and percentage variation of the parameter A of the best-fitting logistic curves of the number of COVID-19 cases and deaths in Italy as a function of the fitting day, March 19–April 13, 2020. (**A**) Trend in the value of parameter A of the curves of the number of cases in Italy and Lombardy. (**B**) Percentage variation of parameter A of the curves of the number of cases and deaths, based on Italian data. (**C**) Percentage variation of parameter A of the curves of the number of cases and deaths, based on data from Lombardy.

Figure 2 A shows the trend in the values of the parameter A of the curve of the number of cases in Italy and Lombardy as a function of the fitting day, from March 19, 2020, when fitting with a logistic curve became statistically relevant (ratio between standard error and saturation value of parameter A: 8% on March 19 versus 51% on March 14), to April 13, 2020. The value of parameter A systematically increased from March 26, 2020, indicating that the observed number of cases was higher than expected based on the logistic curve. As shown in Figure 2B, the percentage variation in the saturation parameter of the curves of cases and deaths based on Italian data was synchronized.

### Case fatality rate and expCFR.

**Figure 3.**
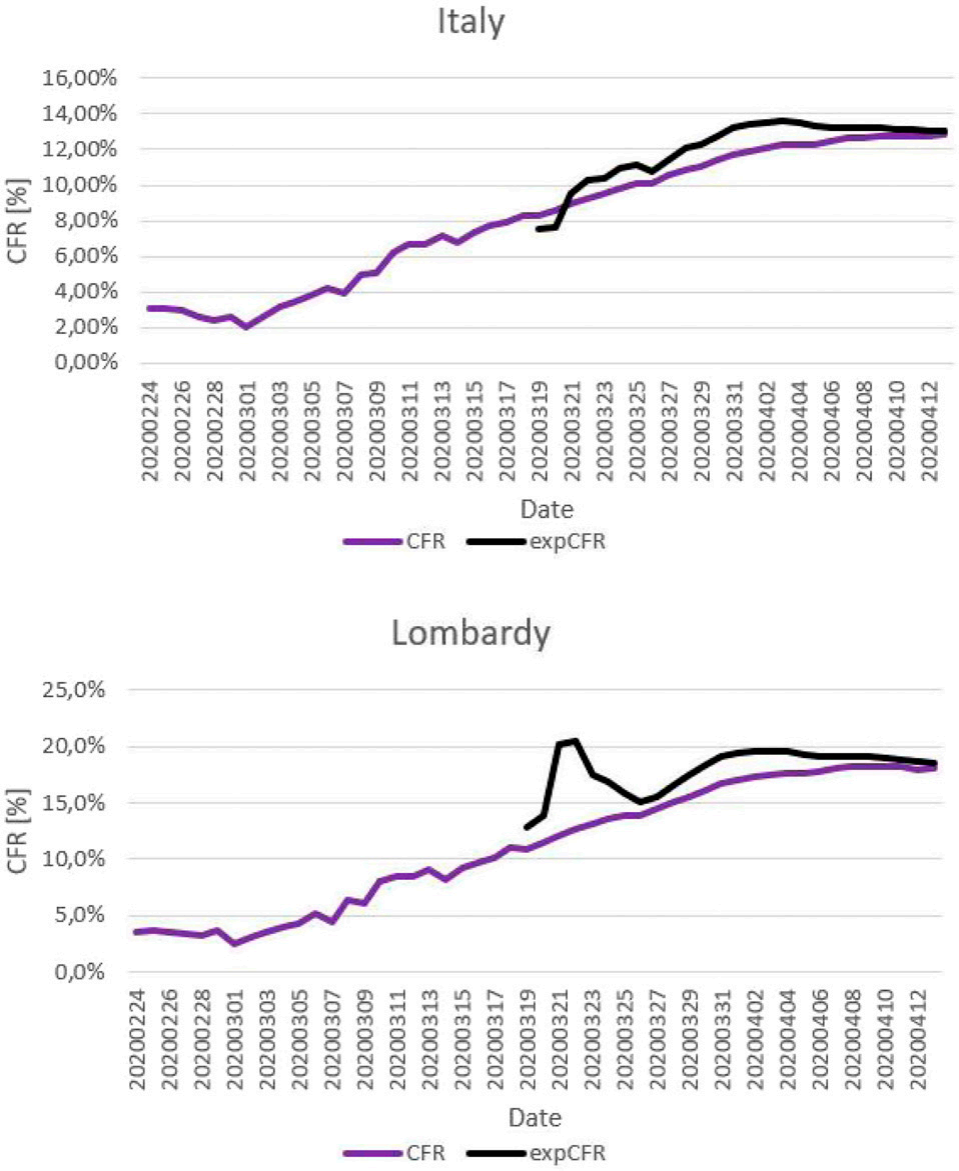
Case fatality rate (CFR) and expected CFR (expCFR) of the 2020 COVID-19 epidemic in Italy and Lombardy as a function of time. (**A**) Case fatality rate^a^ as a function of time and expCFR^b^ as a function of time, Italy. (**B**) Case fatality rate and expCFR as a function of time, Lombardy. ^a^Case fatality rate is calculated by dividing the number of deaths in patients who tested positive for COVID-19 by the cumulative number of cases at a specific point in time. ^b^Expected CFR is calculated by dividing the A parameters of the best-fitting logistic curves of the cumulative number of deaths and cases, at a specific point in time.

Figure 3 shows the CFR (from February 24 to April 13) and the expCFR (from March 19 to April 13, due to statistical relevance) in Italy and Lombardy as a function of time. During the study period, the CFR decreased in the first phase, in which the number of cases was not sufficient for a statistically relevant analysis, and then systematically increased from 2% to 3% on March 2 to 12.8% and 18.1% on April 13, for Italy and Lombardy, respectively. Conversely, the growth rate of the CFR decreased as a function of time because of the decreasing effect of the delay between cases and deaths with the increase in their cumulative numbers. The expCFR reached a plateau and decreased from April 4. The expCFR calculated on April 13 was 13% for Italy and 18.5% for Lombardy.

### TVAR%.

**Figure 4.**
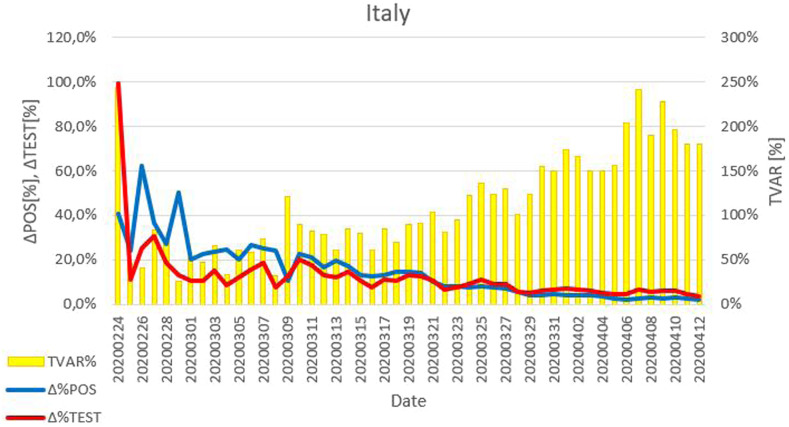
Daily percentage variation of positive results among performed tests (POS%), daily percentage variation of performed tests, and value of TVAR%, that is, daily percentage variation of the number of performed tests, divided by the daily percentage variation of POS%, as function of time, during the 2020 COVID-19 outbreak in Italy.

As shown in Figure 4, from the beginning of the study period until March 24, the daily percentage variation of POS% was higher than the daily percentage variation of the number of performed tests, resulting in a value of TVAR% lower than 100%. After March 25, the value of TVAR% was greater than 100%, indicating that the number of performed tests increased proportionally more than POS%.

### Correlation analysis.

**Figure 5.**
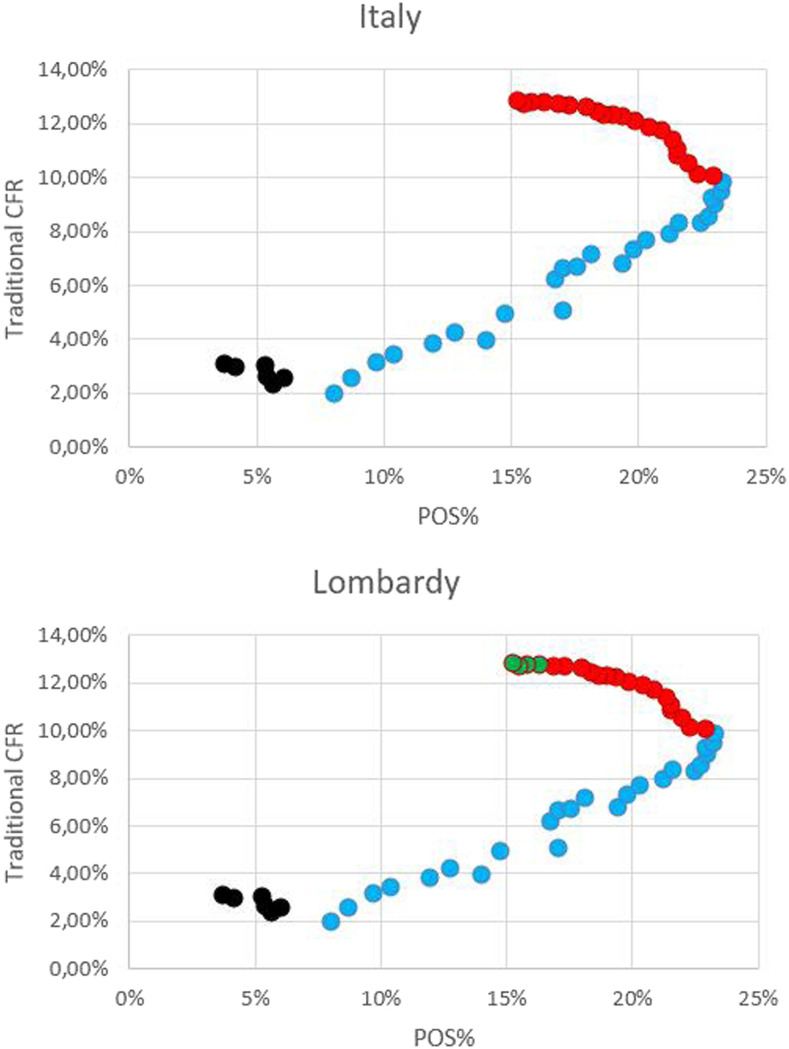
Correlation between the percentage of positive results among performed tests (POS%) and case fatality rate (CFR) during the 2020 COVID-19 outbreak in Italy and Lombardy. (**A**) Correlation between POS% and CFR^a^, based on Italian data. (**B**) Correlation between POS% and CFR^a^, based on data from Lombardy. Black dots: data from February 24 to February 29. Blue dots: March 1–24. Red dots: March 25–April 13. Green dots: April 10–13 (only data from Lombardy). ^a^Case fatality rate is calculated by dividing the number of deaths in patients who tested positive for COVID-19 by the cumulative number of cases at a specific point in time.

**Figure 6.**
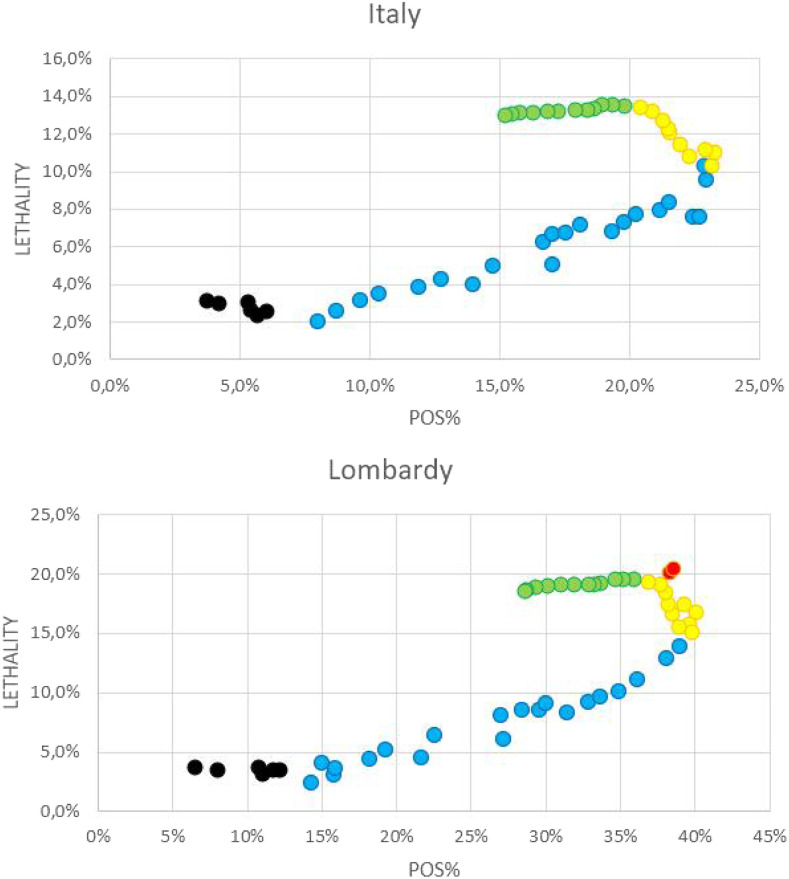
Correlation between the percentage of positive results among performed tests (POS%) and expected fatality during the 2020 COVID-19 outbreak in Italy and Lombardy. (**A**) Correlation between POS% and case fatality rate (CFR)^a^ from February 24 to March 19, and between POS% and expected CFR (expCFR)^b^ from March 19 to April 13, based on data from Italy. (**B**) Correlation between POS% and CFR^a^ from February 24 to March 18, and between POS% and expCFR^b^ from March 19 and April 13, based on data from Lombardy. Black dots: data from February 24 to February 29. Blue dots: March 1–24. Green dots: March 25–April 3. Yellow dots: April 4–13. Red dots: March 21–22, outlier values for CFR that were excluded from the correlation analysis of the data from Lombardy. ^a^Case fatality rate is calculated by dividing the number of deaths in patients who tested positive for COVID-19 by the cumulative number of cases at a specific point in time. ^b^Expected CFR is calculated by dividing the A parameters of the best-fitting logistic curves of the cumulative number of deaths and cases, at a specific point in time.

The scatterplots depicted in Figures 5 and 6 show the correlation between POS% and fatality, and results of the analysis are summarized in Table 2. Analyzing the correlation between POS% and CFR based on data from Italy and Lombardy (Figure 5A and B, respectively), a similar trend was found, and three periods were identified in both plots: 1) data from February 24 to 29, not statistically relevant; 2) data from March 1 to 24, the CFR increased with %POS; and 3) data from March 25 to April 13, the CFR increased whereas POS% decreased. Regarding the second cluster of data, as previously reported, TVAR% was lower than 100% until March 24; therefore, in this phase, there was a growing underestimation of positive cases, which led to a growing overestimation of the CFR. During the third identified period, although POS% decreased as TVAR was greater than 100%, the CFR continued to increase. It is hypothesized that this inconsistency was due to the delay between cases and deaths, which became critical when the curve of cases reached its peak before the curve of deaths did. Considering data from Lombardy, a fourth cluster could be identified: 4) data from April 10 to 13, both POS% and CFR decreased, although the result of the correlation analysis was not statistically significant. As the outbreak in Lombardy preceded the evolution of the epidemic in Italy, the time delay between cases and deaths could have become negligible in this phase because of the low influence of the delay on high cumulative numbers.

**Table 2:** Results of the correlation analysis between the percentage of positive results among performed real-time PCR tests and case fatality rate,* based on data from the 2020 COVID-19 outbreak in Italy and Lombardy

	Italy		Lombardy	
Time period	Correlation coefficient	Average slope (95% CI)	Correlation coefficient	Average slope (95% CI)
February 24–February 29	−0.78	−0.27 (−0.64; 0.11)	−0.46	−0.04 (−0.18; 0.1)
March 1–March 24	0.98†	0.46 (0.42; 0.51)	0.98†	0.38 (0.34; 0.42)
March 25–April 9	−0.92†	−0.35 (−0.43; −0.26)	−0.87†	−0.5 (−0.64; −0.36)
April 10–April 13 (only data from Lombardy)		–	0.87	0.17 (−0.26; 0.6)

* Case fatality rate is calculated by dividing the number of deaths in patients who tested positive for COVID-19 by the cumulative number of cases at a specific point in time.

† *P* < 0.001.

To evaluate the correlation between POS% and fatality without the distortion due to the delay between cases and deaths, a second analysis was conducted, considering the correlation between POS% and CFR between February 24 and March 18, and the correlation between POS% and expCFR between March 19 (when the fitting of the logistic curves became statistically relevant) and April 14 (Figure 6). Table 3 summarizes the results of the analysis. The plots based on data from Italy (Figure 6A) and from the region of Lombardy (Figure 6B) again showed a similar trend, and four clusters of data could be identified in both plots: 1) data from February 24 to 29, not statistically relevant, 2) data from March 1st to 24, fatality increased with %POS; 3) data from March 25 to April 3, %POS decreased while expCFR kept increasing, corresponding to the delay between symptom onset and death and consistent with the median delay reported by the ISS^18^; and 4) data from April 4 to 13, both expCFR and POS% decreased, albeit with a much less steeper slope than that of the ascending phase (1).

**Table 3:** Results of the correlation analysis between the percentage of positive results among performed real-time PCR tests and fatality,* based on data from the 2020 COVID-19 outbreak in Italy and Lombardy

	Italy		Lombardy	
Time period	Correlation coefficient	Average slope (95% CI)	Correlation coefficient	Average slope (95% CI)
February 24–February 29	−0.78	−0.27 (−0.64; 0.11)	−0.46	−0.04 (−0.31; 0.23)
March 1–March 24	0.96†	0.45 (0.38; 0.52)	0.97†	0.39 (0.33; 0.45)
March 25–April 3	−0.94†	−1 (−1.37; −0.64)	−0.82†	−1.19 (−2; −0.37)
April 4–April 13	0.94†	0.11 (0.08; 0.15)	0.95†	0.12 (0.09; 0.16)

* Correlation between POS% and case fatality rate (CFR) from February 24 to March 18, and between POS% and expected CFR (expCFR) from March 19 and April 13. Case fatality rate is calculated by dividing the number of deaths in patients who tested positive for COVID-19 by the cumulative number of cases at a specific point in time. Expected CFR is calculated by dividing the A parameters of the best-fitting logistic curves of the cumulative number of deaths and cases, at a specific point in time. Data from March 21–22 were excluded from the correlation analysis of the data from Lombardy as these were outliers for the CFR.

† *P* < 0.001.

## DISCUSSION

Considering the CFR has a crucial role in informing decisions on the intensity, timing, and duration of public health interventions,^10^ and the unprecedented scale and costs of the mitigation measures implemented in our country,^12^ it is vital that CFR estimates are reliable and based on accurate data.

During the first 50 days of the epidemic in Italy, the crude CFR showed an increasing trend. A significant positive correlation was found between CFR and POS% until March 25, when a sufficient daily increase in the number of tests was achieved. This suggests the surveillance system did not detect a growing number of cases in the initial phase of the epidemic, leading to an increasing underestimation of the CFR. Conversely, after an initial increasing phase, the expCFR reached a plateau and began a downward trend from the 40th day of the epidemic. Furthermore, in the final phase of the correlation analysis, a decrease in both expCFR and POS% could be identified, suggesting an improvement in epidemiologic surveillance. This trend was not found analyzing the crude CFR, indicating the methodology we propose for the calculation of the expCFR could be more accurate, as it is not distorted by the delay between cases and deaths. The analysis of the epidemic in the region of Lombardy, where hospitals endured enormous pressure and reached maximum capacity, showed a similar trend.

According to the results of this study, ascertainment bias could be a more plausible explanation for the trend in fatality, rather than a breakdown of the healthcare system. The high value and increasing trend of the CFR in Italy could be explained by testing strategies and capacity, which were insufficient to detect the true scale of the outbreak until the 40th day of the epidemic. Insufficient testing and isolation of cases could have facilitated the widespread transmission of COVID-19 in the early stages of the outbreak, both in the community and in healthcare facilities.

As shown in Table 4, the predicted values of the total numbers of cases and deaths, interpolated using logistic curves based on data from the first 50 days of the outbreak, are lower than the observed numbers of cases and deaths in Italy as of July 31, 2020. Whereas the logistic curves reached an expected plateau shortly after the end of our study period, the actual number of cases and deaths followed a linear growth for several weeks. This could be due to the important underestimation of cases and deaths in the initial phases of the epidemic, which may have led to biased results in the longer term. A severe underestimation, particularly of mild and asymptomatic cases, was hypothesized analyzing data from the outbreak in China.^19^ The uncertainty of the official Chinese data may have led to inaccurate forecasts by orders of magnitude.^19^ Phylogenetic analysis suggests that the virus could have been spreading for weeks before the first cases were reported in Lombardy, infecting unknown numbers of people.^20^ Furthermore, the percentage of underreported cases in Italy was estimated to be among the highest worldwide.^21^ The number of performed tests progressively improved, as shown by the value of ΔTEST%. Despite the non-negligible divergence between predictions based on the logistic curves and the observed data, the difference between the expCFR calculated based on data from the first 50 days of the epidemic and the CFR calculated based on observed data from July 31, 2020 is minimal (Italy: expCFR = 13%, CFR = 14.2%; Lombardy: expCFR = 18.5%, CFR = 17.5%).This study had several other limitations. First, the definition of deaths related to COVID-19 in Italy requires a positive RT-PCR test, which is not always performed in patients who are not hospitalized or with a rapidly evolving disease. Therefore, a certain degree of ascertainment bias could also affect the numerator of the CFR. Second, the number of performed tests does not correspond to the number of tested individuals, as very often the same individual is tested several times, although not on the same day. Third, notifications of cases and deaths from individual regions are often delayed, leading to important fluctuations in daily increases.

**Table 4:** Estimated values of the A parameters of the best-fitting logistic functions modeling the cumulative number of cases and deaths as a function of time, based on data from the first 50 days of the COVID-19 outbreak in Italy and Lombardy and observed data as of July 31, 2020

		Predicted value (A parameter)	Observed value
Cases	Italy	170,785	247,537
	Lombardy	62,441	96,219
Deaths	Italy	22,193	35,141
	Lombardy	11,559	16,806

Our results are in line with those of a previous analysis of the CFR trend in China and Wuhan, where an upward trend in the initial phase of the outbreak was also found, and increasing ascertainment bias was suggested as a plausible explanation.^10^ The same phenomenon occurred during the SARS epidemic: different countries reported varying estimates, and an increasing trend in the crude CFR was identified. This variation was in retrospect largely explained by difficulties in standardizing definitions of cases and deaths, and the increase in the CFR over time was most likely an artifact due to the estimation method. The possibility that the increase in the CFR could be due to a more lethal mutation of the virus was suggested during the SARS epidemic, causing misinformation and panic, and highlighting the detrimental effect of inconsistent epidemiologic intelligence.^22^

Since June 15, 2020, Italy has begun progressively de-escalating social distancing measures. During this transition phase, accurate estimates of the CFR will be essential to guide the evolution of public health measures. Furthermore, considering no effective vaccine or definitive treatment are available, prompt testing, rigorous contact tracing, and immediate isolation of cases are currently the only option to reduce transmission and prevent a second wave of infections. South Korea, Singapore, Taiwan, and Hong Kong rapidly implemented widespread testing, aggressive contact tracing, and isolation, demonstrating the effectiveness of these strategies without, in the case of South Korea, having to resort to the extreme social distancing measures that were implemented in Italy.^23^ Technologically enhanced contact tracing and isolation of even the mildest cases outside the community may also have contributed to the success of these strategies.^24^

In Italy, until recently the Ministry of Health recommended testing should be prioritized for symptomatic patients already suspected of being infected and requiring hospitalization. The region of Veneto did not apply this stringent policy and used mass testing in the most affected areas, which included asymptomatic and mildly symptomatic individuals.^5^ On April 13, the region of Veneto had tested around 4% of its population, compared with 2% in Lombardy. Applying the same methodology, we estimated that the expCFR based on data from Veneto was 6.4% on April 13, nearly a third of the expCFR in Lombardy estimated on the same date (18.5%). A recent modeling study demonstrated that diagnosis campaigns can decrease both the peak and duration of the epidemic, as identified cases can be isolated, reducing further transmission.^25^ Increasing the identification of cases also allows patients to receive timely clinical care. Results of a study of the epidemic in Veneto suggest that testing asymptomatic and mildly symptomatic patients could reduce the proportion of patients requiring intensive care, which in turn reduces the risk of reaching maximum intensive care capacity.^26^

However, resource constraints limit the ability to perform widespread testing, and it is understandable that in a setting with limited laboratory capacity or during a surge of infections, testing should be rationalized. In China, the category of clinically confirmed cases was introduced for a brief extent of time in the province of Hubei. Patients were included in this category if they have met the clinical criteria and had radiological evidence of viral pneumonia, regardless of epidemiological links or laboratory confirmation of infection. The introduction of this category allowed the timely isolation and treatment of highly suspected hospitalized cases, whereas testing resources could be reallocated for the identification and isolation of cases in the community.^27^

Currently, the gold standard for identifying COVID-19 cases is RT-PCR testing, which is time consuming and requires certified laboratories and expensive equipment.^25^ The development of accurate and reliable rapid tests that could be performed at point-of-care and of validated serological tests to investigate immunity toward COVID-19 will help achieve effective population-wide surveillance.

In conclusion, results of this study suggest data from the first 50 days of the COVID-19 epidemic in Italy were severely affected by ascertainment bias. Resulting epidemiologic parameters such as the CFR should therefore be interpreted with caution. In the post-lockdown phase, more accurate data will be essential to inform policy and avoid second waves of infection, which could be achieved by further expanding testing capacity and broadening case definitions. An important challenge Italy faced during the COVID-19 outbreak was the lack of a strong centralized response. The regionalization of the healthcare system resulted in significant differences in terms of surveillance strategies, mitigation measures, as well as in the clinical management of infected patients among Italian regions, and led to fragmented epidemiological data.^28–30^ The severe course of the COVID-19 epidemic in Italy highlights the urgent need to invest in public health and build up outbreak preparedness in our country, which would allow us to provide rapid, effective, and coordinated responses to future pandemic threats.
